# Jinkui Shenqi Pills Ameliorate Asthma with “Kidney Yang Deficiency” by Enhancing the Function of the Hypothalamic-Pituitary-Adrenal Axis to Regulate T Helper 1/2 Imbalance

**DOI:** 10.1155/2018/7253240

**Published:** 2018-02-08

**Authors:** Bing Ji, Yuan-yuan Li, Wei-ji Yang, Li-zong Zhang, Ming-sun Fang, Hui-ying Fu, Qi-yang Shou

**Affiliations:** ^1^Huzhou Hospital of Traditional Chinese Medicine, Affiliated Zhejiang Chinese Medical University, HuZhou 313003, China; ^2^Institute of Comparative Medicine & Experimental Animal Research Center, Zhejiang Chinese Medical University, Hangzhou 310005, China; ^3^Graduate School, Zhejiang Chinese Medical University, Hangzhou 310053, China; ^4^Second Clinical Medical College, Zhejiang Chinese Medical University, Hangzhou 310005, China

## Abstract

The aim of the study was to investigate the effects and underlying mechanism of JKSQP in a rat model of asthma with kidney-yang deficiency (KYD).* Materials and Methods*. Hydrocortisone (HYD) was used to establish the rat model of KYD; rats were then sensitized and challenged with ovalbumin (OVA). JKSQP was administered to OVA-challenged rats, and the changes in signs and symptoms of KYD were observed. The leukocyte number and subpopulations in bronchoalveolar lavage fluid (BALF) were counted and the cells were stained with Wright–Giemsa dye. Serum adrenocorticotropic hormone (ACTH), corticosterone (CORT), corticotropin-releasing hormone (CRH), total immunoglobulin E (IgE), and OVA-specific IgE levels were determined using relevant enzyme-linked immunosorbent assays (ELISA) kits.* Results*. JKSQP not only reversed the phenomenon of KYD but also significantly inhibited the number of leukocyte and eosinophils in the BALF, increasing the level of interferon (IFN)-*γ* and decreasing the levels of interleukin-4 (IL-4) and IgE in the serum compared with the OVA-challenged groups.* Conclusions*. Taken together, the antiasthma effects of JKSQP were likely mediated by the enhancement of the function of the hypothalamic-pituitary-adrenal axis and the reversal of T helper 1/2 imbalance.

## 1. Introduction

Asthma, which has numerous clinical manifestations, is characterized by chronic eosinophilic inflammation [1, 2]. There are 300 million patients with asthma globally, and China has one of the highest asthma-related death rates. Practitioners of traditional Chinese medicine (TCM) believe that recurrent asthma and its inherent features are closely related to kidney-yang deficiency (KYD). Moreover, poor function of the hypothalamic-pituitary-adrenal axis (HPAA) is commonly reported in patients with allergies, and HPAA dysfunction is a key feature of KYD [3]. The pathogenesis of asthma has not yet been clearly elucidated, but the imbalance in T cell-mediated immune regulation and chronic airway inflammation are deemed the most important mechanisms of asthma development [4, 5]. *β*-2 receptor agonists, glucocorticoids, leukotriene receptor blockers, and combination therapies are currently the main treatments for asthma.

Asthma can be traced back to almost 2000 years ago in ancient China and was first mentioned in Huangdi Neijing (黄帝内经), which also recorded the use of TCM to cure asthma [6]. The Jinkui Shenqi pill (JKSQP, 金*匮肾*气丸) is an important representative formula for the treatment of asthma dispensed by* Zhang Zhongjing* in the Synopsis of Prescriptions of the Golden Chamber (also named* Jin Kui Yao Lue*) and has been widely used to treat KYD syndrome [7–9]. Modern pharmacology research has indicated its effects including lowering blood pressure [7], improving sexual dysfunction [10] and lipid profile, and protecting renal function [11], however these were mainly related to the characteristics of KYD syndrome. Although currently there are only few studies on the antiasthmatic effects of JKSQ pill (Jiang et al., 2015), the mechanism underlying the action of JKSQP in treating asthma has not been understood clearly. Therefore, in this experiment, we explored the relationship between asthma and KYD syndrome as well as the mechanism mediating the curative effects of JKSQP on asthma.

## 2. Material and Methods

### 2.1. Experimental Animals and Groups

Sixty pathogen-free female Wistar rats (10–12-week-old) were purchased from Shanghai Laboratory Animal Center, Chinese Academy of Sciences [production permit: (Hu) 2007-0005]. The animals were acclimatized for 1 week under constant temperature (22°C), humidity (72%), and a 12-h light/dark cycle. The rats had free access to a standard laboratory diet and were provided water ad libitum. Then, the rats were randomly assigned to six groups (*n* = 10 per group): control, ovalbumin (OVA), OVA + JKSQP, hydrocortisone (HYD), HYD + OVA, and HYD + OVA + JKSQP groups.

### 2.2. Drug Test and Treatment

JKSQP (金*匮肾*气丸, batch number 20140112) was produced by Henan Wan-West Pharmaceutical Co., Ltd., (Henan, China). The raw materials consisted of eight herbs: processed aconite (Fuzi, Radix Lateralis Preparata Aconiti Carmichaeli, 9.0 g), Cassia twig (Guizhi, Ramulus Cinnamomi Cassiae, 3.0 g), Rehmannia (Dihuang, Radix Rehmanniae Glutinosae, 24.0 g), Dioscorea root (Shanyao, Dioscoreae Rhizoma, 10.0 g), Cornus fruit (Shanzhuyu, Corni Fructus, 12.0 g), Alisma (Zexie, Rhizoma Alismatis, 9.0 g), Poria (Fuling, Scierotium Poreae Cocos, 9.0 g), and Cortex of the Peony Tree Rote (Danpi, Cortex Radicis Moutan, 9.0 g). The pills were dissolved in water to prepare a solution before the experiment, and the animals were orally given 14 g/kg, which is 10 times the human dose.

**Figure 1:**
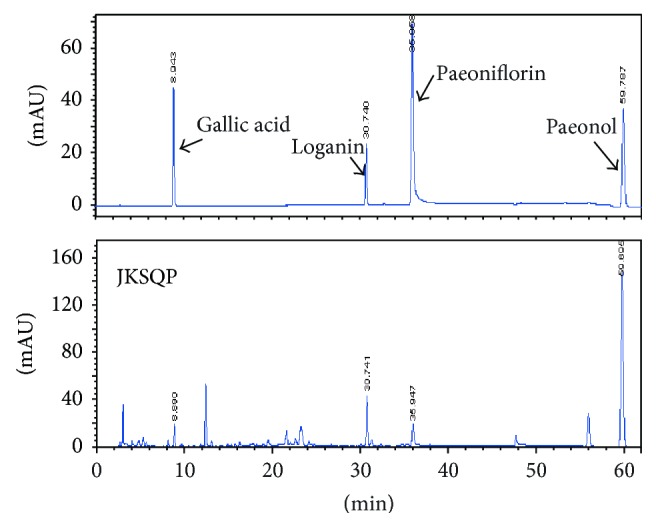
Fingerprints of Jinkui Shenqi pills (JKSQP) detected using high-performance liquid chromatography (HPLC).

The fingerprint of the JKSQP was further analyzed using a high-performance liquid chromatography (HPLC) system. The fingerprints of the mixed standard compounds and JKSQP are shown in Figure 1. The HPLC analyses of JKSQP were performed using the Agilent XDB-ODS column (250 × 4.6 mm, 5-*μ*m diameter). The mobile phase was 0.5% phosphoric acid solution (A) and acetonitrile (B). The following gradient elution mode was used: 0–20 min, 98–89% A; 20–38 min, 89–83% A; 38–43 min, 83% A; 43-44 min, 83–62% A; and 44–62 min, 62% A. The detection wavelengths were 234 nm and 274 nm, the flow rate was 1 mL/min, and the column temperature was 30°C.

### 2.3. KYD Rat Model

The rat KYD model was established by the administration of 15 mg/kg HYD for subcutaneous injections for 20 consecutive days.

### 2.4. Ovalbumin- (OVA-) Induced Asthmatic Model

Seven days after the last HYD injection, the rats were sensitized and challenged with 200 *μ*g/100 *μ*L OVA (grade V, Sigma-Aldrich, St. Louis, MO, USA) mixed with aluminum hydroxide via subcutaneous injections of 0.1 mL into six different parts: the bilateral groin, notum, and hind vola. In addition, they received intraperitoneal injections of 0.4 mL, and the treatments were repeated after 7 days. Two weeks after the sensitization, the rats were given 10 g/L OVA via inhalation for 30 min over the next 7 days.

### 2.5. Observation of Signs and Symptoms

During model establishment, the general state of the animals including body weight, anal temperature, food and water intake, urine volume and color, stool condition, hogback, curl-up, and irritability responses was recorded every 4 days. In the late stage of the experiment, all the rats were kept in a quiet, dark environment for 1 min, and then a multifunction event recorder was used to count their autonomic activity for 5 min.

### 2.6. Measurement of Serum Cytokines

After intraperitoneal injections of sodium pentobarbital, heart blood samples were collected, and the serum was subsequently separated using a refrigerated centrifuge at 4°C and 3000 rpm for 10 min. Levels of adrenocorticotropic hormone (ACTH), corticosterone (CORT), corticotropin-releasing hormone (CRH), total immunoglobulin E (IgE), and OVA-specific IgE were detected using enzyme-linked immunosorbent assay (ELISA) kits.

### 2.7. Bronchoalveolar Lavage and White Blood Cell Count and Classification

Anesthetized rats had undergone endotracheal intubation and bronchial ligation of the right lung preventing them from entering the lavage and then they were injected with Hanks solution containing heparin 1 mL for 3 times from the trachea intubation to the airway. The rinse was collected in the test tube. White blood cell count and classification count: the perfusate was diluted with 1% acetic acid and the total number of white blood cells was counted using a microscope. The perfusate was smeared onto the glass slide, stained with Wright–Giemsa dye, then classified, and counted under high power magnification; calculate the ratio and number of white blood cell subgroup.

### 2.8. Cytokine Detection in Lung Tissue

Lung tissues were weighed, and an equal volume of saline solution was added; the tissues were homogenized and centrifuged at 10000 rpm/min for 10 min. The supernatant was used for relative cytokine detection performed according to the manual of interleukin (IL)-4 and interferon (IFN)-*γ* ELISA kit (R&D System, Minneapolis, MN).

### 2.9. Lung Histology

The right lungs were dissected, fixed in 10% paraformaldehyde overnight at 4°C, and followed by embedment in paraffin and then the tissue samples were cut into 4-*μ*m sections. The sections were heated at 60°C for 2 h and then stained after conventional dewaxing with xylene and washing with ethanol followed by water. Then, the sections were stained with hematoxylin and eosin (H&E) for general morphological analysis and examination of cell infiltration. Both stains were subsequently observed using power field microscopy.

### 2.10. Statistical Analysis

All the data were expressed as means ± standard deviation (SD). Differences between mean values of normally distributed data were assessed using a one-way analysis of variance (ANOVA) using the statistical package for the social sciences (SPSS) 17.0 software. For comparison of two groups, Student's *t*-test and *χ*^2^ test were used and *P* < 0.05 was considered statistically significant.

## 3. Results

### 3.1. Detection of Body Weight, Autonomic Activity, and Body Temperature of Different Groups

**Figure 2:**
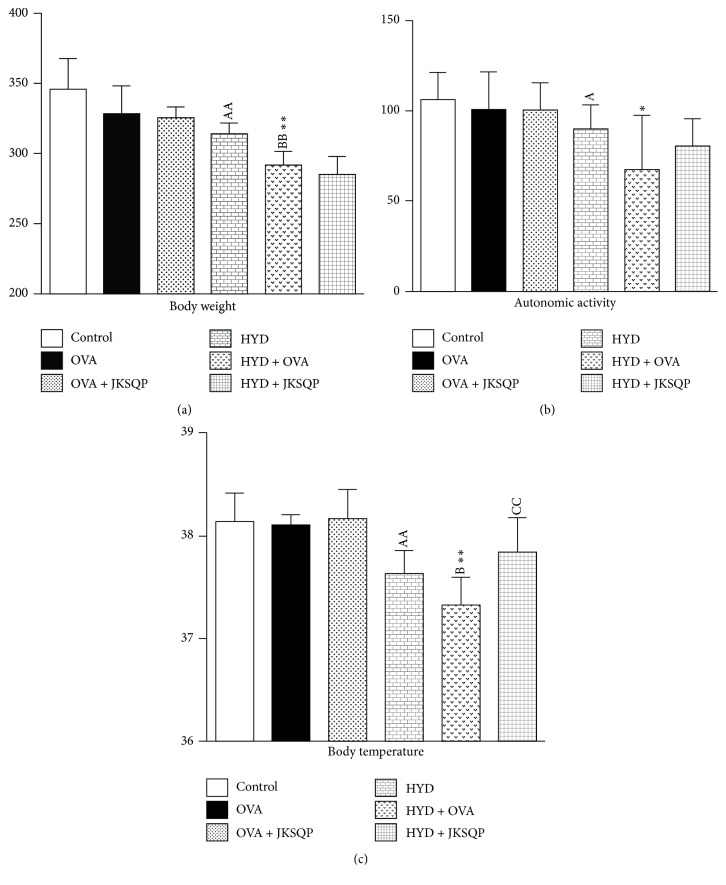
*Symptoms recorded during entire experiment.* No significant difference was reported in body weight, autonomic activity, and body temperature among the six groups before the experiment. (a) Body weight changes of OVA-challenged rats with or without JKSQP treatment. (b) Autonomic activity duration of OVA-challenged rats with or without JKSQP treatment. (c) Body temperature of OVA-challenged rats with or without JKSQP treatment. Data are mean ± standard deviation (SD) of 10 rats per group. ^AA^*P* < 0.01 and ^A^*P* < 0.05 versus control group; ^BB^*P* < 0.01 and ^B^*P* < 0.05 versus HYD group; and ^CC^*P* < 0.01 versus HYD + OVA group; ^*∗∗*^*P* < 0.01 and ^*∗*^*P* < 0.05 versus OVA group. OVA: ovalbumin, HYD: hydrocortisone, and JKSQP: Jinkui Shenqi pills.

First, we determined the differences in body weight among the groups, and as shown in Figure 2(a), the body weight of the HYD group decreased significantly compared with that of the control group (*P* < 0.01); the body weight of OVA-challenged rats with KYD decreased significantly compared with that of the OVA and HYD groups (*P* < 0.01). Second, the autonomic activity duration of the HYD group significantly decreased compared with that of the control group (*P* < 0.05 and *P* < 0.01); the HYD and OVA group also showed a marked decrease in autonomic activity duration compared with the OVA group (Figure 2(b)). Moreover, the anal temperature of the HYD group with or without OVA challenge obviously decreased compared with that of the control group and OVA group, respectively, while JKSQP markedly reversed the decreased body temperature induced by HYD and OVA (*P* < 0.01, see Figure 2(c)).

### 3.2. Detection of Numbers of BALF Leukocyte and Its Subpopulation among Groups

**Figure 3:**
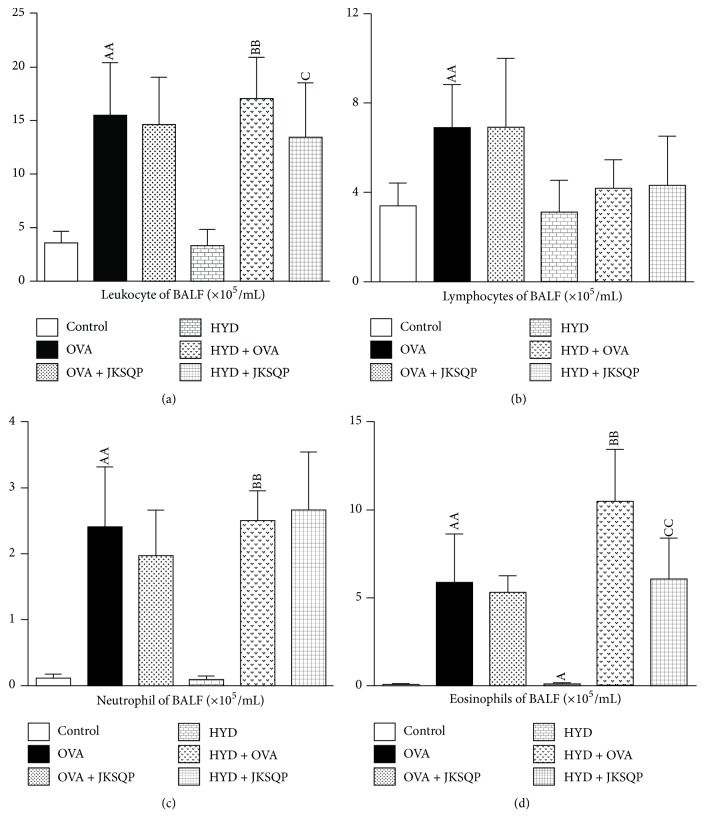
*(a) Leukocyte, (b) lymphocyte, (c) neutrophil, and (d) eosinophil levels in bronchoalveolar lavage fluid (BALF) evaluated at the end of the experiment using enzyme-linked immunosorbent assay (ELISA).* ELISA was performed according to the manufacturer's protocol. Data are mean ± standard deviation (SD) of 10 rats per group. ^AA^*P* < 0.01 and ^A^*P* < 0.05 versus control group; ^BB^*P* < 0.01 versus HYD group; ^CC^*P* < 0.01 and ^C^*P* < 0.05 versus HYD + OVA group. OVA: ovalbumin, HYD: hydrocortisone, and JKSQP: Jinkui Shenqi pills.

The results showed that the OVA challenge significantly increased the number of leukocytes, neutrophils, and eosinophils in the BALF of the OVA group compared with that of the control group (*P* < 0.01), as well as in the HYD + OVA group compared with that of the HYD group (Figure 3). Moreover, the number of eosinophils in the BALF of the HYD + OVA group significantly increased compared with that of the only OVA-challenged group (Figure 3(d)). While JKSQP obviously inhibited the increase in leukocyte and eosinophils numbers in the BALF of OVA-challenged rats with HYD, no effects were observed in the normal OVA-challenged rats (Figures 3(a) and 3(d)).

### 3.3. Evaluation of Serum CORT, ACTH, and ACTH Levels of Rats

**Figure 4:**
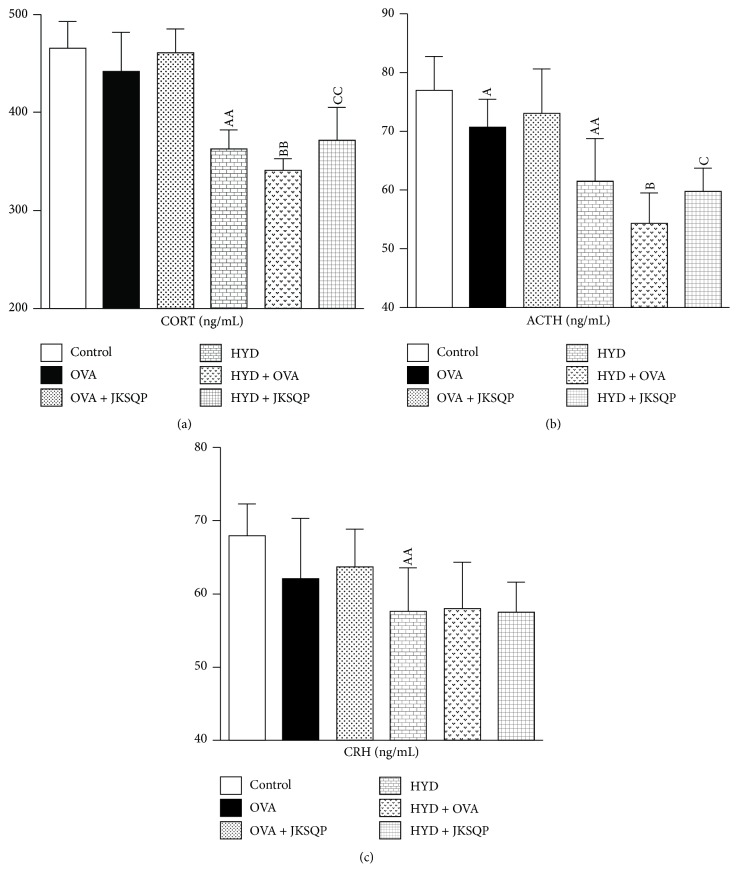
*(a) Serum corticosterone (CORT), (b) adrenocorticotropic hormone (ACTH), and (c) corticotropin-releasing hormone (CRH) evaluated at the end of the experiment using enzyme-linked immunosorbent assay (ELISA)*. ELISA was performed according to the kit manufacturer's protocol. Data are mean ± standard deviation (SD) of 10 rats per group. ^AA^*P* < 0.01 and ^A^*P* < 0.05 versus control group; ^BB^*P* < 0.01 and ^B^*P* < 0.05 versus HYD group; and ^CC^*P* < 0.01 and ^C^*P* < 0.05 versus HYD + OVA group. OVA: ovalbumin, HYD: hydrocortisone, and JKSQP: Jinkui Shenqi pills.

ACTH, CORT, and CRH are widely used as dynamic criteria for evaluating KYD in TCM clinics, and their levels in the serum from the HYD group rats significantly decreased compared with those from the control group rats (*P* < 0.01 and *P* < 0.05). Moreover, OVA challenge more obviously decreased the levels of CORT and ACTH in rats with KYD (Figures 4(a) and 4(b)), but no distinct changes occurred in CORT levels of the normal rats. However, JKSQP reversed these symptoms in OVA-challenged rats with HYD (Figures 4(a) and 4(b)).

### 3.4. Evaluation of T Helper 1 (Th1) and Th2 Cytokines in Rat BALF and Serum

**Figure 5:**
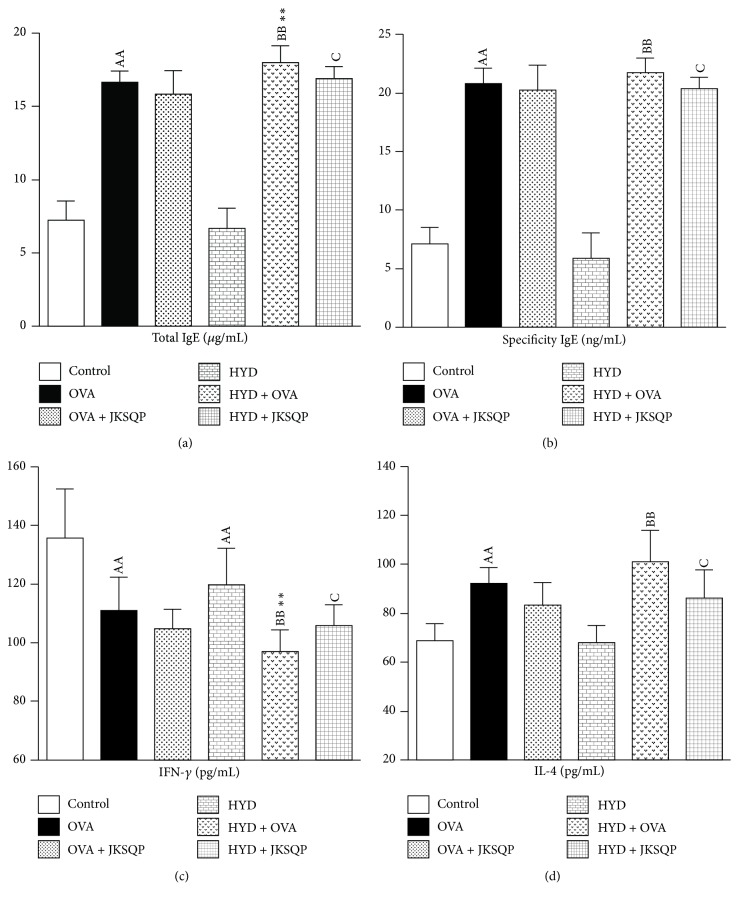
*(a) Serum total immunoglobulin E (IgE) and (b) its specificity, (c) lung interferon (IFN)-γ, and (d) interleukin (IL)-4 levels evaluated at the end of the experiment using enzyme-linked immunosorbent assay (ELISA).* ELISA kits were used according to the manufacturer's protocol. Data are mean ± standard deviation (SD) of 10 rats per group. ^AA^*P* < 0.01 versus control group; ^BB^*P* < 0.01 versus HYD group; ^C^*P* < 0.05 versus HYD + OVA group; ^*∗∗*^*P* < 0.01 versus OVA group. OVA: ovalbumin, HYD: hydrocortisone, and JKSQP: Jinkui Shenqi pills.

The total IgE levels of the various groups are shown in Figure 5 A, which distinctly revealed that the total IgE content in OVA-induced asthmatic rat serum significantly increased compared with that in the normal group (*P* < 0.01), as well as in the HYD + OVA group compared with that of the HYD group. Furthermore, allergen-specific IgE is believed to be inextricably associated with the induction of allergic airway symptoms and, therefore, is used as a guide for environmental modification and immunotherapy. The results shown in Figure 5(b) indicate that rats without or with KYD sensitized with OVA had a significantly increased IgE level compared with that of the normal and HYD groups, respectively (*P* < 0.01). However, JKSQP inhibited the increase in total IgE and their specific IgE levels in OVA-challenged rats with KYD (Figures 5(a) and 5(b)).

Interferon (IFN)-*γ* and interleukin (IL)-4, which have been shown to be the crucial cytokines in the serum and involved in the pathogenesis of asthma, are reported in patients with atopy. Compared with that in the normal group, the concentration of IFN-*γ* in the OVA group significantly decreased following the induction of asthma (*P* < 0.01). In addition, the IL-4 concentration was increased by OVA exposure compared with that in the unexposed normal group (*P* < 0.01), as well as in the HYD + OVA group compared with that in the HYD group. JKSQP reversed these changes by increasing the level of IFN-*γ* and decreasing that of IL-4 (Figures 5(c) and 5(d)).

### 3.5. Lung Histopathological Analysis

**Figure 6:**
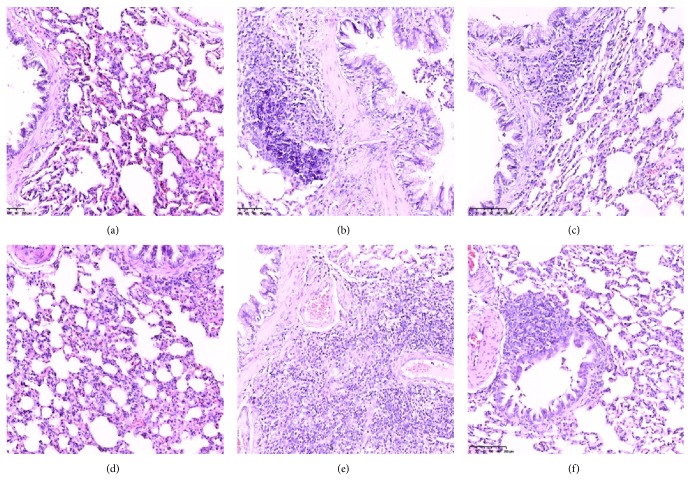
*Histological examinations of lung tissues for inflammatory cell infiltration.* Lung tissues obtained at the end of the experiment were stained with hematoxylin and eosin (H&E, 200x magnification). Groups: (a) Control, (b) OVA, (c) OVA + JKSQP, (d) HYD, (e) HYD + OVA, and (f) HYD + OVA + JKSQP. OVA: ovalbumin; HYD: hydrocortisone; JKSQP: Jinkui Shenqi pills.

The characteristic features of asthmatic airways are cell inflammation, the presence of hyperplastic goblet cells, mucus secretion, and collagen deposition [12]. The results in Figure 6 clearly show that the histological sections of lung tissue from the normal group had no detectable inflammatory response in the alveolar, bronchial, or vascular walls. However, lung tissue from the asthma and KYD asthmatic groups exhibited increased mucous plug obstruction and inflammatory secretions in the bronchial lumen and extensive infiltration of inflammatory cells around the airways and blood vessels, especially eosinophils and lymphocytes.

Moreover, hyperplastic bronchi, vascular smooth muscle, and a wide variability in alveolar interval were observed in the OVA-challenged rat lungs. This evidence indicates that the KYD model rats were more susceptible to developing OVA-induced asthma than the normal rats. Conversely, airway inflammation was inhibited more in the histological sections of lung tissue from the JKSQP-treated rats than it was in sections from the KYD plus OVA groups. These results were consistent with the score of HE staining (Table 1); for scoring criteria, please see Ji et al., 2005.

## 4. Discussion

The theory of TCM proposes that the physiological function of the body would be at a low level in KYD syndrome [13]. In this study, we observed that the KYD rat model exhibited lower autonomous activities and anal temperature than the normal group. The levels of CRH, ACTH, and CORT more significantly decreased in the KYD groups than they did in the normal group. Furthermore, HPAA hypofunction was observed in the KYD rat model. These results were similar to those observed in a specific KYD animal model in a previously reported study [3, 14].

Th1/Th2 imbalance, especially the excessive expression of Th2 cytokines, is a critical mechanism involved in mediating asthma attacks. The function of Th1 cytokines decreases, while that of Th2 markedly increases in patients with asthma [15]. The Th2 cytokine IL-4 induces B cells to differentiate into plasma cells, which then produce IgE [16, 17]. Eventually, the development of allergy is mainly associated with eosinophil infiltration and chronic airway inflammation, which rely on IgE. Moreover, IL-4 not only promotes the differentiation of Th0 cells to Th2 cells but also inhibits the effect of Th1 cells. In contrast, IFN-*γ* inhibits the synthesis of IgE by B cells while it suppresses Th0 cell differentiation into Th2 cells. Furthermore, IFN-*γ* inhibits the agglomeration of eosinophils in the airway [18, 19].

From the test data, the level of the Th1 cytokine, IFN-*γ*, in the KYD groups decreased more than that in the normal group, which subsequently increased the level of the Th2 cytokine, IL-4. The Th1/Th2 ratio was altered in the KYD groups [20], while the KYD + OVA group showed a more significant increase in the levels of leukocytes, eosinophils, and total and specific IgE than the OVA group did. Moreover, the HPAA was hypofunctional in the KYD model, which decreased the physiological secretion of glucocorticoid. This suggests that a patient with KYD would likely experience asthma attacks more easily than a normal individual with the same risk factor. These results prove a close relationship between KYD and asthma and provide evidence supporting the “warming” of KYD in treating asthma.

JKSQP was recorded in the* Jingui Yaolue *(*Synopsis of Golden Chamber*,* 金匮要略*), which was written by Zhang Zhongjing at the end of the Eastern Han Dynasty 1800 years ago. JKSQP was considered one of the best kidney-yang warming drugs and has been widely used to treat numerous diseases affecting various body systems such as chronic diarrhea, edema, and especially asthma, which TCM doctors have treated with this agent for thousands of years. More than 200 years ago,* Huangdi Neijing *(*黄帝内经*), the earliest and greatest medical classic extant work on the physiology and pathophysiology of TCM in China, reported a close relationship between kidney-yang and asthma. Furthermore, Zhu Danxi considered phlegm an important factor in the pathogenesis of asthma. KYD is an important mechanism involved in the formation of phlegm [21, 22] and, therefore, JKSQP is widely used to treat asthma.

As mentioned above, HPAA hypofunction and Th1/Th2 imbalance were observed in all OVA groups, while JKSQP treatment showed improvements in these parameters. Furthermore, the leukocyte, eosinophil, and total and specific IgE levels, which are biomarkers of airway inflammation, were lower in the asthma and drug groups than in the two OVA groups. In addition, the lesions in the asthmatic drug-treated lung tissues improved more than those in the two OVA groups did. These effects may be attributable to the JKSQP-induced improvement of HPAA function, which also increased glucocorticoid secretion.

Furthermore, the increased glucocorticoid secretion inhibited the expression of Th2 cytokine subpopulations. Then, the inflammation induced downstream by Th2 cytokines would reduce, thereby improving the airway inflammation and reducing the hyperreaction, which would control the asthmatic attack. However, other mechanisms might be involved in the antiasthmatic effect induced by treatment with JKSQP, in addition to the pathway associated with improved HPAA function, which reverses the imbalance in Th1/Th2. These speculations are worth further investigation in future studies.

## 5. Conclusion

KYD is associated with HPAA hypofunction and Th1/Th2 imbalance and is a risk factor that affects and exacerbates asthma. JKSQP cures and controls asthma by improving the HPAA function and reversing the imbalance between Th1 and Th2 cytokines, which inhibits or reduces the airway inflammation. Therefore, this study provides evidence to support the effectiveness of JKSQP in the treatment of asthma with KYD.

## Figures and Tables

**Table 1:** The score of HE staining for all groups.

	Peripheral blood vessels and bronchial EOS increase	Edema	Epithelial cell injury
Control	0.16 ± 0.40	0	0
OVA	3.33 ± 0.51^AA^	3.00 ± 0.63^AA^	2.66 ± 0.51^AA^
OVA + JKSQP	3.1 ± 0.75	2.83 ± 0.75	2.50 ± 0.54
HYD	0.33 ± 0.51	0	0
HYD + OVA	4.83 ± 0.41^BBB^	4.50 ± 0.83^BBB^	4.33 ± 0.81^BBB^
HYD + OVA + JKSQP	3.50 ± 0.83^CC^	3.33 ± 0.51^CC^	3.16 ± 0.41^CC^

^AA^ *P* < 0.01 versus control group; ^CC^*P* < 0.01 versus HYD + OVA group; ^BBB^*P* < 0.001 versus HYD group. OVA: ovalbumin, HYD: hydrocortisone, and JKSQP: Jinkui Shenqi pills.

## Abbreviations

ACTH: Adrenocorticotropic hormone
BALF: Bronchoalveolar lavage fluid
CORT: Corticosterone
CRH: Corticotropin-releasing hormone
ELISA: Enzyme-linked immunosorbent assays
H&E: Hematoxylin and eosin
HYD: Hydrocortisone
HPAA: Hypothalamic-pituitary-adrenal axis
IgE: Immunoglobulin E
IFN: Interferon
IL: Interleukin
JKSQP: Jinkui Shenqi pills
KYD: Kidney-yang deficiency
OVA: Ovalbumin
TCM: Traditional Chinese medicine.
